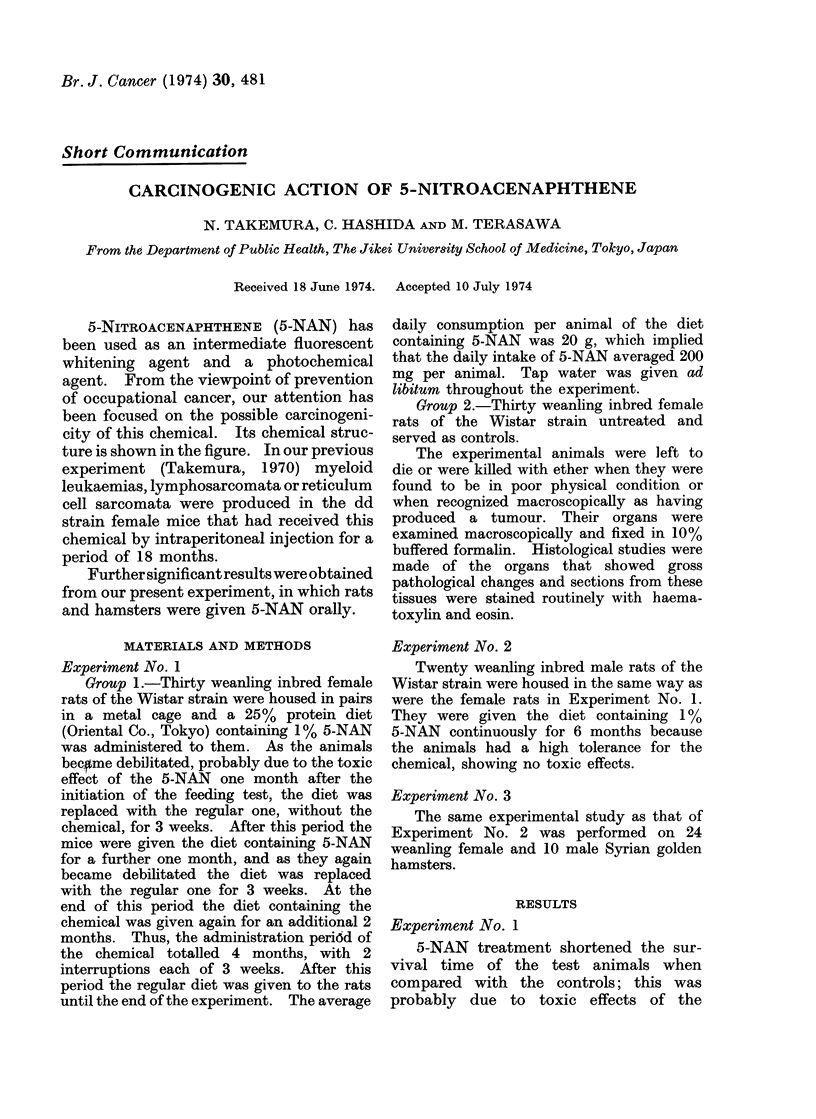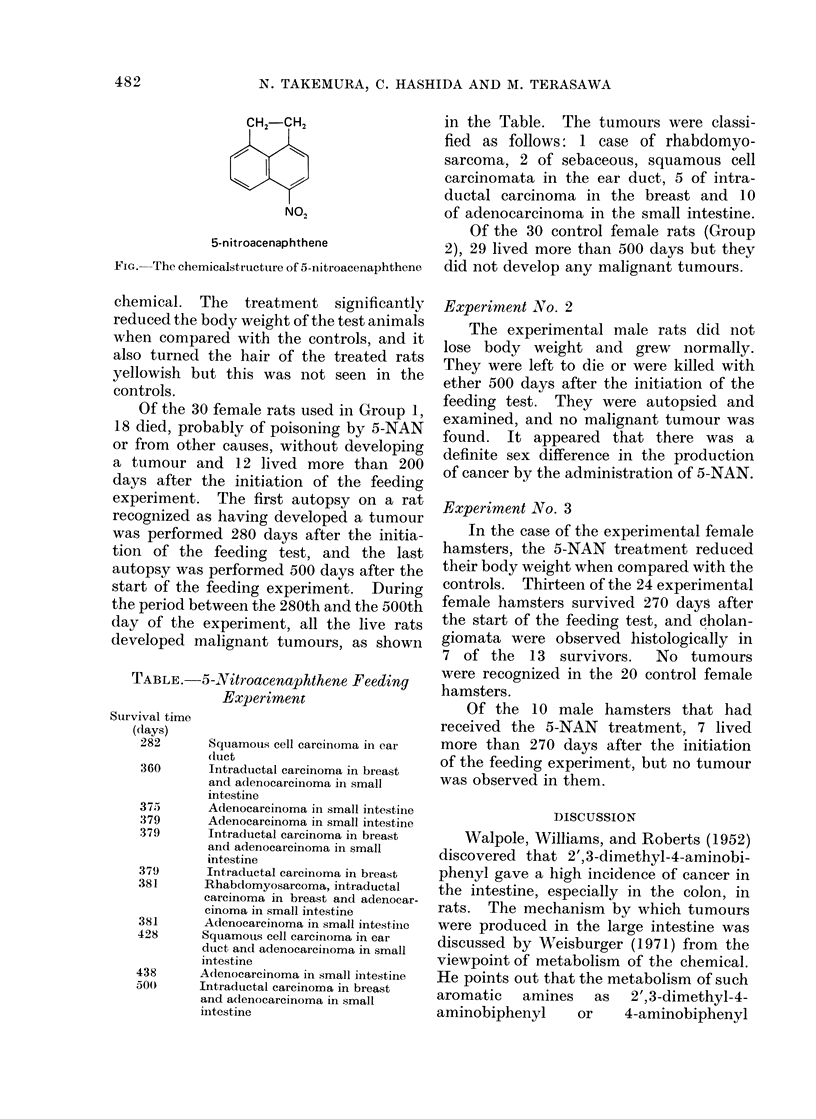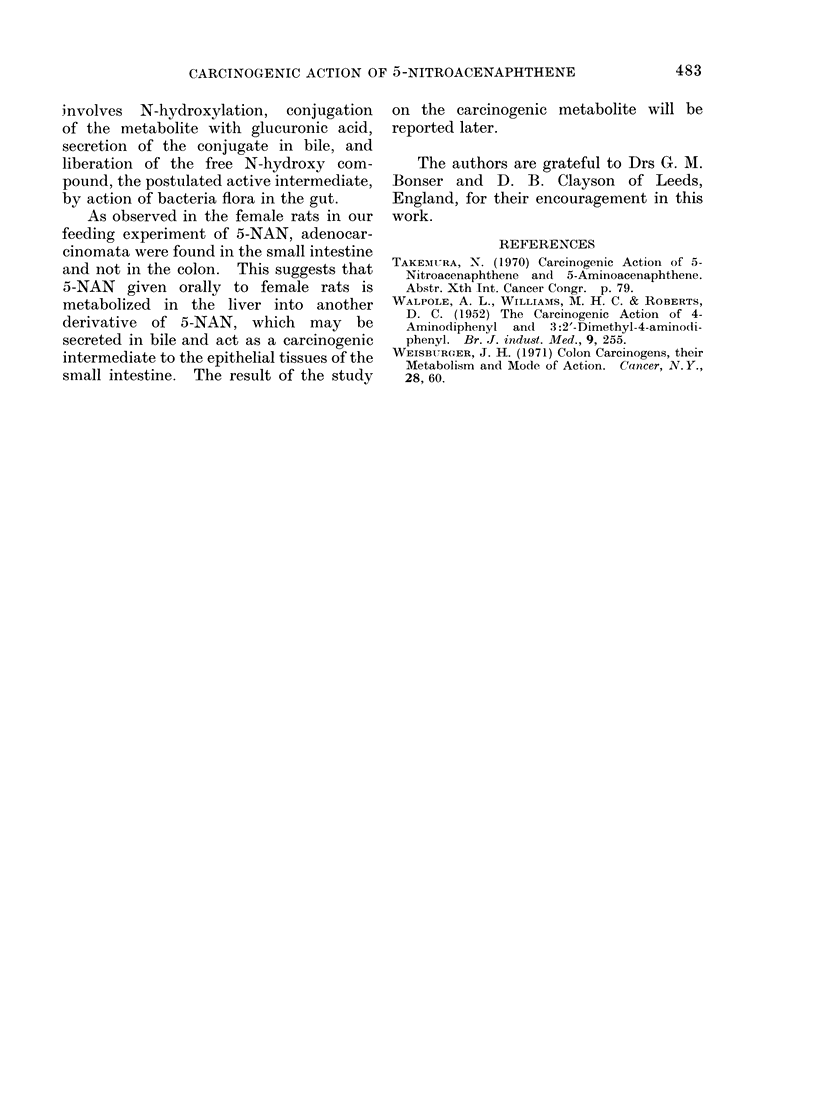# Carcinogenic action of 5-nitroacenaphthene.

**DOI:** 10.1038/bjc.1974.225

**Published:** 1974-11

**Authors:** N. Takemura, C. Hashida, M. Terasawa


*Br. J. Cancer* (1974) **30**, 481

***Short Communication***

### CARCINOGENIC ACTION OF 5-NITROACENAPHTHENE

N. TAKEMURA, C. HASHIDA AND M. TERASAWA

*From the Department of Public Health, The Jikei University School of Medicine, Tokyo, Japan*

Received 18 June 1974. Accepted 10 July 1974

5-NITROACENAPHTHENE (5-NAN) has been used as an intermediate fluorescent whitening agent and a photochemical agent. From the viewpoint of prevention of occupational cancer, our attention has been focused on the possible carcinogenicity of this chemical. Its chemical structure is shown in the figure. In our previous experiment (Takemura, 1970) myeloid leukaemias, lymphosarcomata or reticulum cell sarcomata were produced in the dd strain female mice that had received this chemical by intraperitoneal injection for a period of 18 months.

Further significant results were obtained from our present experiment, in which rats and hamsters were given 5-NAN orally.

### MATERIALS AND METHODS

*Experiment No.* 1

*Group* 1.—Thirty weanling inbred female rats of the Wistar strain were housed in pairs in a metal cage and a 25% protein diet (Oriental Co., Tokyo) containing 1% 5-NAN was administered to them. As the animals became debilitated, probably due to the toxic effect of the 5-NAN one month after the initiation of the feeding test, the diet was replaced with the regular one, without the chemical, for 3 weeks. After this period the mice were given the diet containing 5-NAN for a further one month, and as they again became debilitated the diet was replaced with the regular one for 3 weeks. At the end of this period the diet containing the chemical was given again for an additional 2 months. Thus, the administration period of the chemical totalled 4 months, with 2 interruptions each of 3 weeks. After this period the regular diet was given to the rats until the end of the experiment. The average daily consumption per animal of the diet containing 5-NAN was 20 g, which implied that the daily intake of 5-NAN averaged 200 mg per animal. Tap water was given *ad libitum* throughout the experiment.

*Group* 2.—Thirty weanling inbred female rats of the Wistar strain untreated and served as controls.

The experimental animals were left to die or were killed with ether when they were found to be in poor physical condition or when recognized macroscopically as having produced a tumour. Their organs were examined macroscopically and fixed in 10% buffered formalin. Histological studies were made of the organs that showed gross pathological changes and sections from these tissues were stained routinely with haematoxylin and eosin.

*Experiment No.* 2

Twenty weanling inbred male rats of the Wistar strain were housed in the same way as were the female rats in Experiment No. 1. They were given the diet containing 1% 5-NAN continuously for 6 months because the animals had a high tolerance for the chemical, showing no toxic effects.

*Experiment No.* 3

The same experimental study as that of Experiment No. 2 was performed on 24 weanling female and 10 male Syrian golden hamsters.

### RESULTS

*Experiment No.* 1

5-NAN treatment shortened the survival time of the test animals when compared with the controls; this was probably due to toxic effects of the

5-nitroacenaphthene

FIG.—The chemicalstructure of 5-nitroacenaphthene chemical. The treatment significantly reduced the body weight of the test animals when compared with the controls, and it also turned the hair of the treated rats yellowish but this was not seen in the controls.

Of the 30 female rats used in Group 1, 18 died, probably of poisoning by 5-NAN or from other causes, without developing a tumour and 12 lived more than 200 days after the initiation of the feeding experiment. The first autopsy on a rat recognized as having developed a tumour was performed 280 days after the initiation of the feeding test, and the last autopsy was performed 500 days after the start of the feeding experiment. During the period between the 280th and the 500th day of the experiment, all the live rats developed malignant tumours, as shown in the Table. The tumours were classified as follows: 1 case of rhabdomyosarcoma, 2 of sebaceous, squamous cell carcinomata in the ear duct, 5 of intraductal carcinoma in the breast and 10 of adenocarcinoma in the small intestine.

Of the 30 control female rats (Group 2), 29 lived more than 500 days but they did not develop any malignant tumours.

TABLE.—*5-Nitroacenaphthene Feeding Experiment*

| Survival time (days) | |
|---|---|
| 282 | Squamous cell carcinoma in ear duct |
| 360 | Intraductal carcinoma in breast and adenocarcinoma in small intestine |
| 375 | Adenocarcinoma in small intestine |
| 379 | Adenocarcinoma in small intestine |
| 379 | Intraductal carcinoma in breast and adenocarcinoma in small intestine |
| 379 | Intraductal carcinoma in breast |
| 381 | Rhabdomyosarcoma, intraductal carcinoma in breast and adenocarcinoma in small intestine |
| 381 | Adenocarcinoma in small intestine |
| 428 | Squamous cell carcinoma in ear duct and adenocarcinoma in small intestine |
| 438 | Adenocarcinoma in small intestine |
| 500 | Intraductal carcinoma in breast and adenocarcinoma in small intestine |

*Experiment No.* 2

The experimental male rats did not lose body weight and grew normally. They were left to die or were killed with ether 500 days after the initiation of the feeding test. They were autopsied and examined, and no malignant tumour was found. It appeared that there was a definite sex difference in the production of cancer by the administration of 5-NAN.

*Experiment No.* 3

In the case of the experimental female hamsters, the 5-NAN treatment reduced their body weight when compared with the controls. Thirteen of the 24 experimental female hamsters survived 270 days after the start of the feeding test, and cholangiomata were observed histologically in 7 of the 13 survivors. No tumours were recognized in the 20 control female hamsters.

Of the 10 male hamsters that had received the 5-NAN treatment, 7 lived more than 270 days after the initiation of the feeding experiment, but no tumour was observed in them.

### DISCUSSION

Walpole, Williams, and Roberts (1952) discovered that 2′,3-dimethyl-4-aminobiphenyl gave a high incidence of cancer in the intestine, especially in the colon, in rats. The mechanism by which tumours were produced in the large intestine was discussed by Weisburger (1971) from the viewpoint of metabolism of the chemical. He points out that the metabolism of such aromatic amines as 2′,3-dimethyl-4-aminobiphenyl or 4-aminobiphenyl involves N-hydroxylation, conjugation of the metabolite with glucuronic acid, secretion of the conjugate in bile, and liberation of the free N-hydroxy compound, the postulated active intermediate, by action of bacteria flora in the gut.

As observed in the female rats in our feeding experiment of 5-NAN, adenocarcinomata were found in the small intestine and not in the colon. This suggests that 5-NAN given orally to female rats is metabolized in the liver into another derivative of 5-NAN, which may be secreted in bile and act as a carcinogenic intermediate to the epithelial tissues of the small intestine. The result of the study on the carcinogenic metabolite will be reported later.

The authors are grateful to Drs G. M. Bonser and D. B. Clayson of Leeds, England, for their encouragement in this work.

### REFERENCES

TAKEMURA, N. (1970) Carcinogenic Action of 5-Nitroacenaphthene and 5-Aminoacenaphthene. Abstr. Xth Int. Cancer Congr. p. 79.

WALPOLE, A. L., WILLIAMS, M. H. C. & ROBERTS, D. C. (1952) The Carcinogenic Action of 4-Aminodiphenyl and 3:2′-Dimethyl-4-aminodiphenyl. *Br. J. indust. Med.*, **9**, 255.

WEISBURGER, J. H. (1971) Colon Carcinogens, their Metabolism and Mode of Action. *Cancer, N.Y.*, **28**, 60.